# Dual variants of uncertain significance in a case of hyper-IgM syndrome: implications for diagnosis and management

**DOI:** 10.3389/fimmu.2025.1594636

**Published:** 2025-06-02

**Authors:** Nourhen Agrebi, Rafah Mackeh, Mohamed Alsabbagh, Asha Elmi, Amnah A. Al-Marri, Satanay Z. Hubrack, Saleema C. Purayil, Mohammed Yousuf Karim, Amel Hassan, Bernice Lo

**Affiliations:** ^1^ Translational Medicine Department, Research Department, Sidra Medicine, Doha, Qatar; ^2^ Allergy & Immunology Division, Department of Medicine, Hamad Medical Corporation, Doha, Qatar; ^3^ Hematology, Immunology and Transfusion Division, Sidra Medicine, Doha, Qatar; ^4^ College of Medicine, Qatar University, Doha, Qatar; ^5^ Pediatric Allergy and Immunology Department, Sidra Medicine, Doha, Qatar; ^6^ College of Health and Life Sciences, Hamad Bin Khalifa University, Doha, Qatar

**Keywords:** *AICDA*, diagnosis strategies, hyper-IgM syndrome, *IKBKB*, VUS

## Abstract

**Background:**

Hyper-IgM syndrome (HIGM) is a genetic immunodeficiency characterized by elevated to normal IgM levels and decreased IgG, IgA, and IgE. The overlapping clinical presentations of different gene mutations complicate diagnosis and management.

**Objective:**

This study aims to elucidate the clinical implications of concurrent *AICDA* and *IKBKB* homozygous variants in a pediatric patient diagnosed with hyper-IgM syndrome.

**Methods:**

We present immunological and genetic analysis of a Tunisian patient with two homozygous variants of uncertain significance (VUSs) in the *IKBKB* and *AICDA* genes, suspected of causing hyper-IgM and immune deficiency. We conducted functional tests to ascertain the pathogenicity of *IKBKB* and *AICDA* mutations and to provide a definitive diagnosis and appropriate management.

**Results:**

Genetic analysis identified two homozygous variants: *AICDA* (p.W80S) and *IKBKB* (p.R77Q). Immunophenotyping and functional studies found greatly reduced class-switched memory B cells and somatic hypermutations but normal T cell responses and NFkB activation.

**Conclusion:**

The simultaneous presence of multiple homozygous VUSs emphasizes a major challenge in the genetic diagnosis of highly consanguinous patients. Functional workup as well as familial segregation studies are needed to clarify variant pathogenicity and provide a definitive diagnosis and tailored treatment strategies for these patients. Our studies suggest that the *AICDA* p.W80S variant is pathogenic, while the *IKBKB* p.R77Q variant is likely benign.

## 1 Introduction

Hyper-IgM syndrome is a rare genetic disorder resulting from B and, in some cases, T cell defects (1). Mutations in the activation-induced cytidine deaminase (*AICDA)* gene lead to autosomal recessive hyper-IgM syndrome type 2 (HIGM2) due to AID deficiency (2, 3). AID plays a crucial role in immunoglobulin class switch recombination (CSR) and somatic hypermutation (SHM) (2). Affected patients often present with recurrent bacterial infections, inflammation, gastrointestinal (GI) infections, lymphoid hyperplasia, and, in some cases, autoimmune cytopenias. However, they typically do not develop opportunistic infections and respond well to human immunoglobulin replacement therapy (4, 5).

Patients with AID deficiency have normal or elevated levels of IgM with low levels of IgG, IgA, and IgE, along with an intact T-cell immunity (1, 2). However, some patients with an increase in IgM and a decrease in other immunoglobulins have been reported with defects in NF-κB pathway components (such as NEMO and IKKβ) (6, 7). For example, Nielsen and colleagues reported a patient with features resembling hyper-IgM but was discovered to have IKKβ deficiency. This report illustrated how it can be challenging to distinguish between these disorders when diagnosing patients due to overlapping clinical presentations (7).

The IKKβ subunit is one of the two catalytic subunits of the IKK complex, which plays a central role in the NF-κB signaling pathway (8). The IKKβ subunit is responsible for phosphorylating the IκB proteins, leading to their degradation and the release of NF-κB for transcriptional activation (9). Inhibition or deficiency of the IKKβ subunit in the NF-κB signaling pathway leads to impaired IκBα phosphorylation and the subsequent inhibition of NF-κB activation (10). Patients with IKKβ deficiency do not respond sufficiently to treatment involving prophylactic antibiotic therapy or human immunoglobulin replacement. They generally require urgent hematopoietic stem cell transplantation (11). It is therefore important to carefully evaluate whether a patient has IKKβ deficiency versus other Inborn Errors of Immunity (IEI).

Herein, we describe a patient of consanguineous parents suspected of having hyper-IgM syndrome. Clinical genetic testing identified homozygous variants of uncertain significance (VUS) in two separate genes, in which pathogenic mutations cause two different IEIs (AID deficiency and IKKβ deficiency). This case led to further evaluation of the patient’s immune phenotype and the functional consequences of each variant to determine which variant might underlie the clinical presentation. Overall the study highlights the importance of functional studies in clarifying the pathogenicity of variants in IEI genes.

## 2 Materials and methods

### 2.1 Human subjects

This study was approved by the Sidra Medicine ethics committee (IRB protocol 1601002512). Informed consent was obtained from all individual participants included in the study and/or their parents.

### 2.2 Activation of T cells with anti-CD3 and anti-CD28 antibodies

Peripheral blood mononuclear cells (PBMCs) were purified by Ficoll density gradient centrifugation. PBMCs were stimulated with 1 μg/mL of anti-CD3 and anti-CD28 antibodies. The cells were cultured 3 days in complete RPMI-1640 medium with 10% FBS at 37°C with 5% CO_2_. Then the cells were cultured with 100 U/mL hIL-2 in fresh media, and fresh hIL-2-containing medium was supplemented every 2 days.

### 2.3 Establishment of EBV-transformed lymphoblasts cell culture

PBMCs of 1-2 million in 1 ml of RPMI 1640 medium with 20% FBS were cultured with cyclosporin A and B95-8 culture supernatant. Every following week, complete RPMI 1640 media with 20% FBS was added. After 3-5 weeks, once lymphoblast proliferation was established, the media was replaced with complete RPMI 1640 media with 10% FBS every two days for continued expansion of the cells or cryopreserved for subsequent culture and expansion.

### 2.4 DNA sequencing

Genomic DNA from blood of the patient was submitted to Invitae and sequenced using their Primary Immunodeficiency Panel, which included 207 genes at the time of analysis. The Invitae diagnostic testing results only found two variants as homozygous. The other VUS were heterozygous and not relevant to the disease phenotype. Familial segregation analysis was conducted by Sanger sequencing of these two homozygous variants. The following primers were used (*IKBKB*-F 5’-gggctctgtcttcctctgtt-3’, *IKBKB*-R 5’-gatctgagaggcaaagcagc-3’; *AICDA*-F 5’-ccagagccgcaataaaagtc-3’, *AICDA*-R 5’-gccttgtctctgagccattc-3’). *IKBKB* and *AICDA* PCRs were carried out using Taq PCR Master Mix Kit (Qiagen) on genomic DNA obtained from activated T cells and EBV-transformed lymphoblasts, respectively.

### 2.5 Isotype-switched memory B cells and numbers of CD45RO-positive memory T cells

PBMCs were stained in 1X PBS 0.3% BSA with appropriate antibodies for analysis of isotype-switched memory B cells (APC-CY7 anti-CD19, PE-Cy7 anti-CD27, FITC anti-IgM, PE anti-IgD) and CD45RO-BV605-positive memory T cells (FITC anti-CD3, APC anti-CD4, APC-CY7 anti-CD8). Flow cytometry data were acquired on the NovoCyte (ACEA Biosciences) and analyzed with FlowJo version 10.

### 2.6 Regulatory T cells levels

Cells were stained with surface marker antibodies (PE anti-CD25, FITC anti-CD3, and AF647 anti-CD127) for 45 minutes on ice. The cells were then washed, fixed/permeabilized using the eBioscience FoxP3 Transcription Factor Staining Buffer kit (ThermoFisher Scientific) and incubated overnight with V450 anti-CD4 and PE-Cy7 anti-FoxP3 (eBioscience) antibodies. Data were analyzed by FlowJo software version 10.

### 2.7 T cell proliferation

PBMCs were incubated with 1 µM of CFSE, washed, and then cultured with beads conjugated to a combination of anti-CD28, -CD3, and -CD2 antibodies (T cell activation/expansion kit, Miltenyi Biotec) for 3 to 5 days. On days 3 and 5, cells underwent staining using Viability dye (LIVE/DEAD™ Fixable Near-IR Dead Cell Stain Kit, ThermoFisher), V450 anti-CD4- and BV605 anti-CD8 (BD Biosciences) antibodies and analyzed on the flow cytometer.

### 2.8 Cytokines

Activated T cells were expanded in 100U/mL IL-2 and restimulated using 20 nM phorbol 12-myristate 13-acetate (PMA) and 1 mM ionomycin (Sigma-Aldrich) for 5 hours at 37°C. After the first hour of stimulation, Brefeldin-A (Invitrogen) was added to block cytokine secretion. After 5-hour stimulation, cells were washed and surface Fc receptors (Biolegend) were blocked for 15 min at 4°C. Cells were stained with APC-Cy7 anti-CD8, then fixed with BD Cytofix/Cytoperm (BD Bioscience) and stained with AF488 anti-CD4, PE anti-IFNγ, BV650 anti-TNFα, and APC anti-IL-2 (eBioscience).

### 2.9 Western blot

For AID protein expression, 2x10^6^ EBV-transformed lymphoblasts were harvested, lysed in hot 1% SDS lysis buffer and sonicated. For IκBα and IKKβ protein analysis, 1x10^6^ activated T cells were lysed. Both lysates were subjected to SDS-PAGE using NuPAGE 4–12% Bis-Tris Gels (Invitrogen). Primary antibodies used included AID monoclonal antibody (#4949S, Cell Signaling Technology), recombinant Anti-IKK beta antibody (ab124957, Abcam), recombinant Anti-IκBα antibody (#4814, Cell Signaling Technology) and Anti-β-Actin Rabbit Polyclonal Antibody (HRP) (VWR).

For the analysis of NFκB pathway proteins, anti-CD3/CD28 activated T cells of the patient, their two siblings, and two healthy unrelated controls (HDs) were expanded in the presence of IL-2 for at least 8 days in Advanced RPMI medium (ThermoFisher #12633012) supplemented with fetal bovine serum L-glutamine, streptomycin, and penicillin. 5x10^5^ cells of each sample were stimulated with 20 ng/mL TNFα (Peprotech #300-01A) for the indicated times, after which the cells were washed twice with ice cold phosphate buffered saline. The samples were lysed in boiling lysis buffer containing 1% SDS 0.1 M Tris-HCl (pH 8.0) and then sonicated. The samples were resolved on 10% polyacrylamide gels at 140 V and transferred onto a PVDF membrane at 110 V for 1 hour on ice. The membrane was blocked with 5% milk in TBST and incubated with anti-phospho-p65 (Cell Signaling #3033) and anti-IκBα antibodies (Cell Signaling #4814) in 3% BSA TBST at 4°C overnight and then with goat anti-mouse (ThermoFisher #A-31553) and anti-rabbit (ThermoFisher #G-21234) peroxidase-conjugated secondary antibody (SeraCare #5220-0341) for 1 hour at room temperature. The membrane was treated with Pierce ECL Western Blotting Substrate (ThermoFisher #32106) and developed using ChemiDoc MP Imaging System (Bio-Rad).

### 2.10 TOPO TA cloning and sequencing of V3-23-Cμ transcripts

Total RNA was extracted from EBV-transformed lymphoblasts generated from the patient, her siblings, and unrelated healthy donors (HDs), including age-matched control. cDNA synthesis was performed as previously described (2, 12) with a Cμ A (5′-GAGGCAGCTCAGCAATC-3′) primer. PCR was performed with the following primers: V3-23 leader exon (5′-GGCTGAGCTGGCTTTTTCTTGTGG-3′) and Cμ B (5′-TCACAGGAGACGAGGGGGAA-3′) (2) (95°C, 5 min; 35 cycles *(94°C, 30sec; 68°C, 30sec; 72°C 45 sec); 72°C, 20 min). PCR products were cloned using the TOPO TA cloning kit (Invitrogen) and V3-23 positive colonies were sequenced. Following manual quality verification of the sequencing chromatograms using UGENE software to confirm single peaks and minimal read length of 292 bp, IgH sequences were aligned to germline sequence (NG_001019.6) and analyzed by Igblast using the default settings to calculate sequence similarity (13). Mutational Frequency was calculated as = 100 - (the number of mutations were divided by the number of nucleotides in the V3-23 region).

### 2.11 Statistical analysis

The statistical analysis was conducted using GraphPad Prism 9 with Mann-Whitney test.

## 3 Results

### 3.1 Clinical and laboratory findings

The present case is a Tunisian patient from consanguineous parents. She was seen aged 9 months in the emergency department with an episode of oral thrush and upper respiratory tract infection, but not admitted to hospital. She was first admitted to the pediatric ward, aged 2 years, with a history of fever, cough and shortness of breath. She was found to have bilateral pneumonia on chest X-ray. She deteriorated clinically and radiologically, and developed hypoxia with respiratory distress despite antibiotic treatment. She was admitted to the Pediatric Intensive Care Unit, treated with oxygen therapy, but did not need ventilation. She also had abdominal pain, constipation, and mild hepatomegaly was reported on ultrasound scan. She was found to have microcytic anemia, and noted to have failure to thrive - her weight was on the 3^rd^ centile. Her microbiology was positive for respiratory syncytial virus, cytomegalovirus (CMV), and serology was IgM+ for herpes simplex virus. Extensive clinical laboratory evaluation revealed marked hypogammaglobulinemia with elevated IgM levels, high CRP, and high vitamin B12. Cellular immunological investigation was performed, and the percentage of T, B and NK cells were within age-adjusted ranges, while memory CD45RO+ cells were elevated for age (**Table 1**). She was within normal limits for lymphocyte proliferative responses to PHA, pokeweed and concanavalin A mitogens.

**Table 1:** Patient characteristics.

Detail	Value (unit)	Normal range
WBC	18.214 x10^3^/mcL	5.0-15.0
RBC	3.5 x10^6^/mcL	4.0-5.2
Hgb	7.0 gm/dL	11.0-14.0
Hct	21.10%	34.0-40.0
MCV	59.6 fL	75.0-87.0
MCH	19.8 pg	24.0-30.0
MCHC	33.2 gm/dL	31.0-37.0
RDW-CV	18.20%	11.6-14.5
Platelet	465 x10^3^/mcL	200-490
MPV	9.7 fL	7.4-10.4
PDW	10.1 fL	9.4-10.6
ANC	14.3 x10^3^/mcL	1.5-8.0
Lymphocyte	2.6 x10^3^/mcL	6.0-9.0
Monocyte	0.8 x10^3^/mcL	0.2-1.0
Eosinophil	0.0 x10^3^/mcL	0.1-1.0
Basophil	0.08 x10^3^/mcL	0.02-0.10
Neutrophil %	80.50%	
Lymphocyte %	14.70%	
Monocyte %	4.20%	
CD3	64.40 %	43.00-76.00
CD3 Absolute Count	2,698.00 cells/mcL	900.00-4,500.00
CD3+/CD4+ %	27.50%	23.00-48.00
CD3+/CD4+ Absolute Count	1,153.00 cells/mcL	500.00-2,400.00
CD3+/CD8+ %	34.00%	14.00-33.00
CD3+/CD8+ Absolute Count	1,426.00 cells/mcL	300.00-1,600.00
CD19	26.60%	14.00-44.00
CD19 Absolute Count	1,117.00 cells/mcL	200.00-2,100.00
CD3-/CD16+/CD56+ %	6.40%	4.00-23.00
CD3-/CD16+/CD56+ Absolute Count	266.00 cells/mcL	100.00-1,000.00
CD4:CD8 Ratio	0.81%	
CD45RA	50.40%	53.00-86.00
CD45RO	40.20%	9.00-26.00
IgG	<0.40 gm/L	4.51-12.02
IgA	<0.05 gm/L	0.15-1.11
IgM	>6.50 gm/L	0.35-1.84
IgG Sub 1	95.9 mg/dL	265.0-938.0
IgG Sub 2	8.7 mg/dL	28.0-216.0
IgG Sub 3	0.2 mg/dL	8.7-86.4
IgG Sub 4	0.3 mg/dL	0.9-74.2
LPA-PHA	Normal	
LPA-ConA	Normal	
LPA-Pok	Normal	
Iron	2 mmol/L	5-18
TIBC	40 mmol/L	45-80
Transferrin	1.6 gm/L	2.0-3.6
Fe% Saturation	5.00%	15-45

WBC, White Blood Cell Count; RBC, Red Blood Cell Count; Hgb, Hemoglobin; Hct, Hematocrit; MCV, Mean Corpuscular Volume; MCH, Mean Corpuscular Hemoglobin; MCHC, Mean Corpuscular Hemoglobin Concentration; RDW-CV, Red Cell Distribution Width - Coefficient of Variation; MPV, Mean Platelet Volume; PDW, Platelet Distribution Width; ANC, Absolute Neutrophil Count; LPA-PHA, Lymphocyte Proliferation Aassay with PhytoHemAgglutinin; LPA-ConA, Lymphocyte Proliferation Assay with Concanavalin A; LPA-Pok, Lymphocyte Proliferation Assay with Pokeweed mitogen; TIBC, Total Iron Binding Capacity.

Based on these results, monthly intravenous immunoglobulin (IVIG) replacement therapy was commenced. Although the initial hepatic ultrasound showed mild hepatomegaly, this was repeated during follow-up and was normal. Liver function tests were performed and showed no evidence of transaminitis or elevated bilirubin. CMV PCR was positive at low levels initially (526 IU/mL), and then subsequently became negative within 6 weeks. The anemia was microcytic, and predominantly due to iron deficiency based on ferritin, transferrin saturation, and blood film appearances. The hemoglobin improved with oral iron supplementation. The patient’s overall condition improved with treatment, and she remains clinically stable under regular follow-up. She has not had any recurrent infections since commencement of IVIG, i.e. over 5 years of follow-up. She, her parents, and 2 sisters had an episode of COVID-19 infection in 2022. She receives trimethoprim/sulphamethoxazole prophylaxis twice daily, three times per week, and the family have been advised on the precautions against cryptosporidium infection.

The patient has dizygotic twin siblings, who are two years older and both currently in good health. One of them (Sibling-2) previously had poor weight gain and history of recurrent minor infections according to the mother. However, these infections did not require admission, and weight gain has improved with age.

### 3.2 Mutations detection

Due to the presence of marked hypogammaglobulinemia with elevated IgM, a genetic panel for primary immunodeficiencies was ordered to investigate a possible underlying inborn error of immunity. Clinical genetic testing using the Invitae PID panel identified two homozygous missense variants of uncertain significance (VUS) in two candidate genes: *IKBKB* c.230G>A, p.R77Q and *AICDA* c.239G>C, p.W80S. Familial segregation analysis by Sanger sequencing of the variants confirmed that the proband was homozygous for the *IKBKB* p.R77Q variant whereas both parents were heterozygous carriers, one sibling was homozygous for the reference allele, and the second sibling whose history included repeated infections and low weight gain was also homozygous for the p.R77Q variant. *IKBKB* encodes for IKKβ, and this mutation, which is located in the kinase domain, changes the IKKβ amino acid residue 77 from arginine, a basic and polar amino acid, to glutamine, a neutral and polar amino acid (p.Arg77Gln). According to the literature and population databases, this variant was novel at the time and has since been reported as a VUS in ClinVar.

Sanger sequencing also confirmed that the proband was homozygous for the *AICDA* p.W80S missense mutation, which changed an aromatic residue to a polar, non-charged residue. Both parents and siblings were heterozygous carriers. The mutation is in exon 3 and affects an amino acid within AID’s catalytic domain known to impact both CSR and SHM.

**Figure 1:**
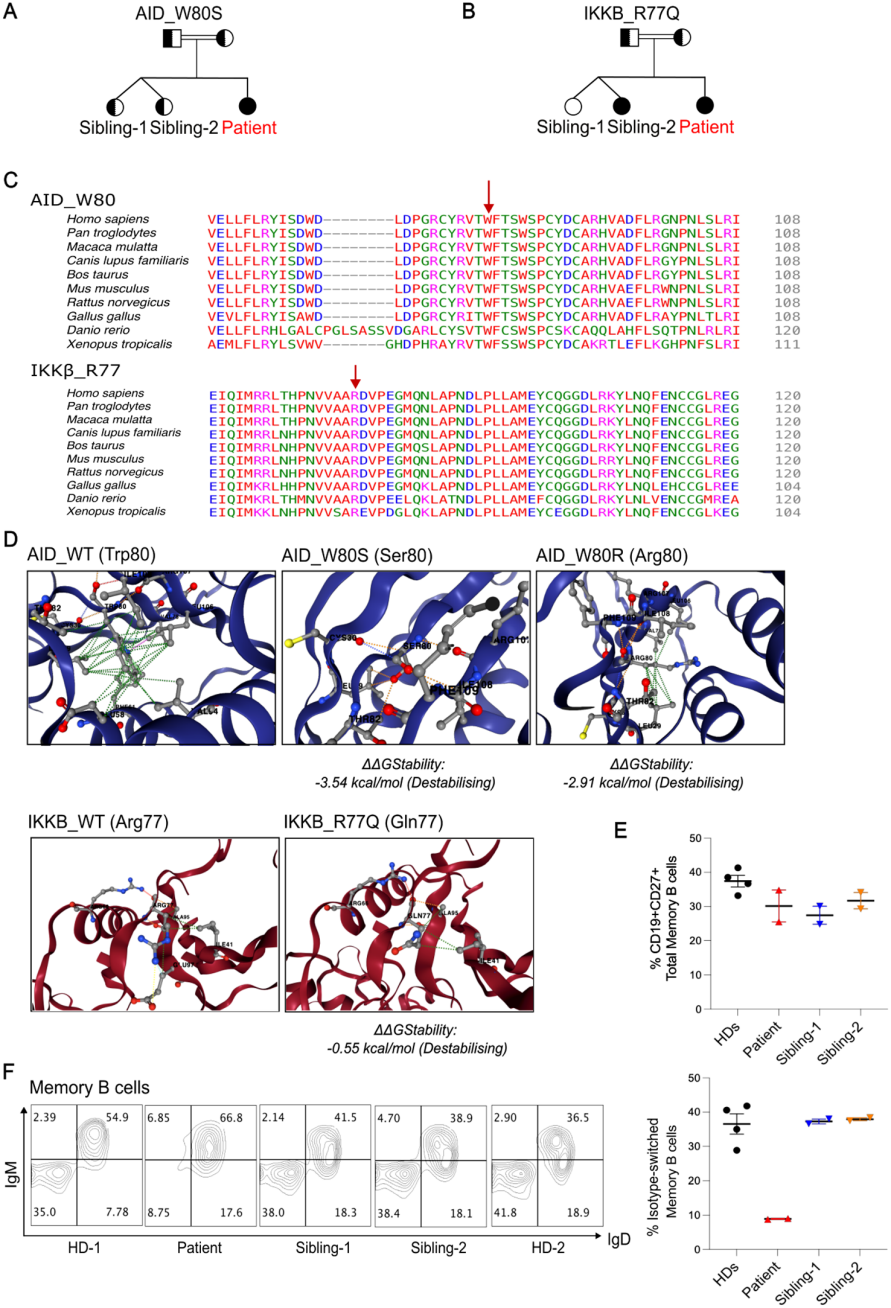
Two homozygous VUSs were identified in a hyper-IgM patient with reduced class-switched memory B cells. **(A**,**B),** Family pedigree showing genotypes of the indicated variants (full shaded = homozygous; half-shaded = heterozygous). **(C)**, Multiple protein alignment demonstrating the degree of conservation of the residues AID p.W80 and IKKb p.R77 in several species. **(D)**, Predicted stability change (ΔΔGStability) results of the genetic variants using DynaMut2. The AID p.W80R mutation was included as a disease control. **(E)**, Quantification of total memory B cell percentages comparing Patient, Siblings and Healthy donors (HDs) from two independent experiments. **(F)**, Representative flow plots showing percentages of non-switched and switched memory B cells within CD19^+^CD27^+^ B cells. Right panel, Quantification of class-switched memory B cells from 2 independent experiments. Class-switched memory B cells within CD19+CD27+ B cells normal range for patient age is 21.9-98.6%.

Pedigree structures for both mutations are shown in **Figure 1** (**Figures 1A, B**).

### 3.3 *In silico* characterization of identified mutations

Both variants were classified as VUS, and several *in silico* variant effect prediction tools indicated that both variants might be deleterious. AID p.W80 and IKKβ p.R77 residues were highly conserved between different species (**Figure 1C**). For the AID p.W80S variant, CADD score was 29.9, PolyPhen-2 score was 1.00 (probably damaging), SIFT score was 0.005 (damaging), MutationTaster predicted disease causing, and PrimateAI score was 0.9045 (damaging). For the IKKβ p.R77Q variant, CADD score was 25.4, PolyPhen score was 0.951 (possibly damaging), SIFT score was 0.336 (tolerated), MutationTaster predicted disease causing, and PrimateAI score was 0.5646 (damaging).

When comparing the wild-type proteins of AID and IKKβ to their mutant forms, structure-based prediction of the variants’ effect on protein stability using DYNAMUT2 revealed a folding free energy change for both structures (**Figure 1D**). Interestingly, we observed that AID p.W80S exhibits higher impact on the stability than the previously reported AID p.W80R mutation (14) (**Figure 1D**).

### 3.4 Class-switched memory B cells

A flow cytometric assessment of peripheral blood lymphocytes revealed normal percentage of total CD27^+^CD19^+^ memory B cells but a reduction of class-switched CD27^+^IgM^-^IgD^-^ memory B cells compared to healthy donors (HDs) and siblings (**Figures 1E, F**).

### 3.5 Functional characterization of the IKKβ R77Q substitution

**Figure 2:**
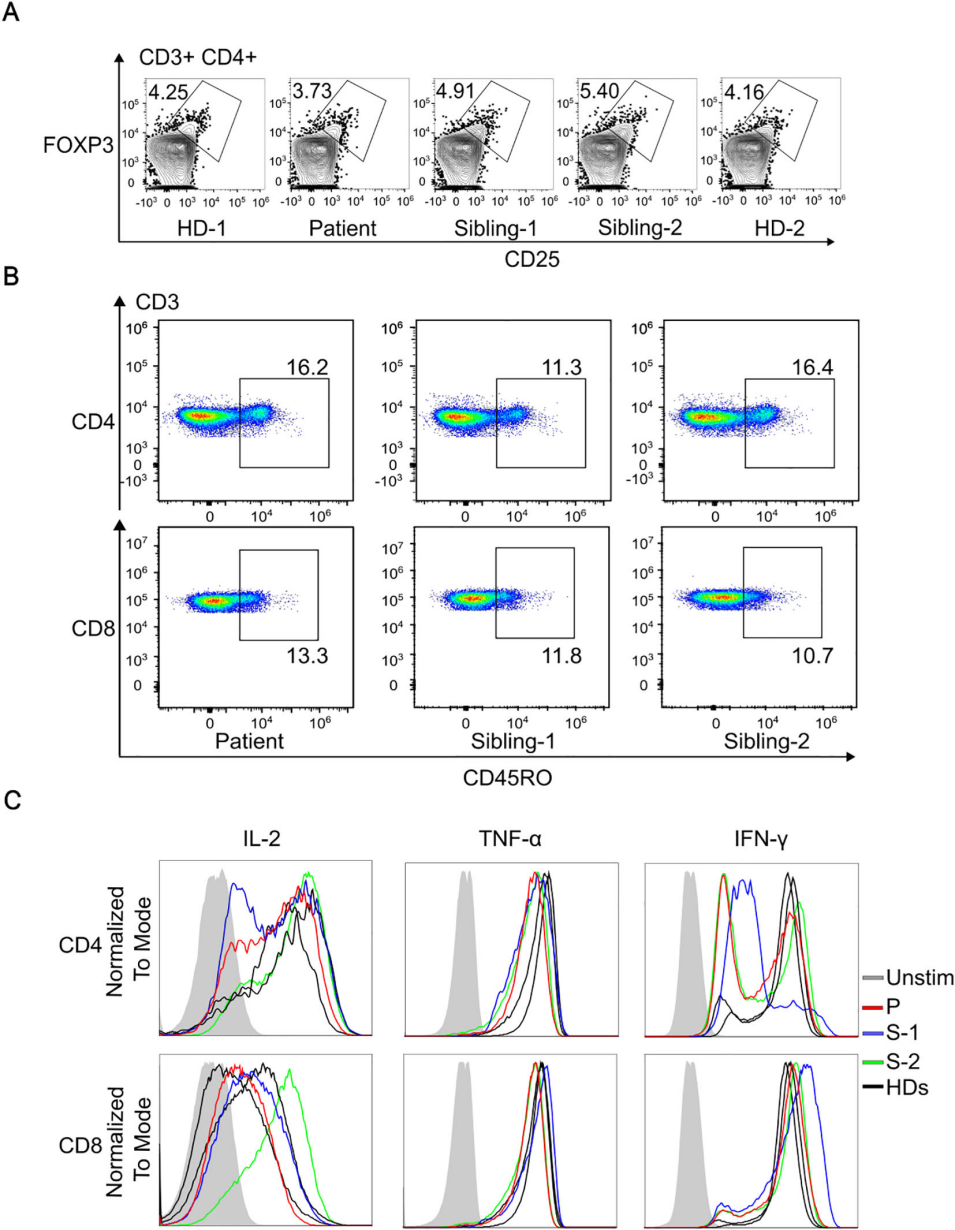
Immunophenotyping of T cells and assessment of IKKb expression. **(A)**, Representative dot plots gated on CD3^+^CD4^+^ cells, illustrating the percentage of Tregs co-expressing CD25 and FoxP3. **(B)**, Comparison of CD45RO+ frequency in CD4+ and in CD8+ T cells in patient and siblings. **(C)**, Representative histogram of flow cytometry showing normal cytokine production comparing the patient, siblings and two healthy donors (HDs).

To investigate potential links between the R77Q IKKβ variant and a biological defect, we assessed Treg and memory T cell levels and function. We found a similar level of CD4^+^CD25^+^FOXP3^+^ Tregs in the patient compared to HDs (**Figure 2A**). We also observed no difference in frequency of CD45RO^+^ memory T cells between the proband and the siblings (**Figure 2B**). Evaluation of T cell cytokine production revealed a normal secretion of IL-2, TNFα and IFNγ (**Figure 2C**).

**Figure 3:**
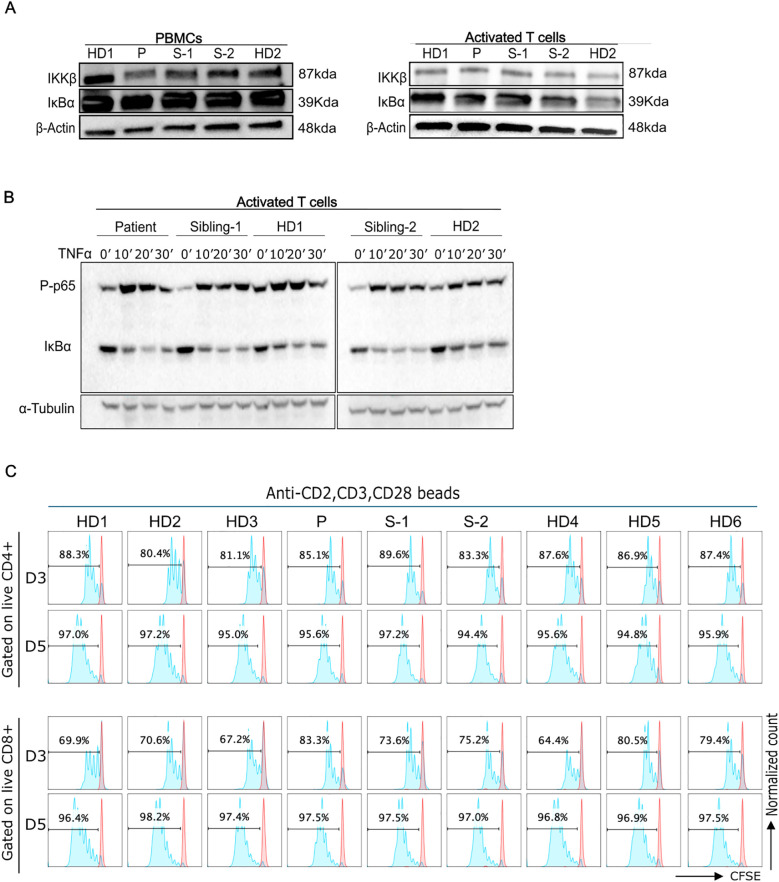
Functional characterizations of *IKBKB* p.R77Q variant. **(A)**, Western blot analysis of PBMCs from the patient showing a lower level of the IKKβ protein (left panel). Protein levels of IkBα were normal as compared with control PBMCs. Right panel, Western blot analysis of activated T cells cultured for 6 days in the presence of IL-2 demonstrating normal levels of the IKKβ and IkBα proteins. Equal amounts of total protein derived from the lysed cells were loaded. **(B)**, Levels of p65 phosphorylation and IkBα degradation at different time points (0-30min) post-TNFa stimulation. Left blots are representative of 3 independent experiments, and right blots are representative of 2 independent experiments. **(C)**, Representative CFSE cell proliferation profiles for T cells are shown for days 3 and 5 as indicated for CD4 and CD8 T cells. Numbers above gate indicate the percent of cells divided in response to activation with anti-CD2, CD3, and CD28 beads. Data are representative of 4 independent experiments.

We assessed the IKKβ and IκBα protein levels in PBMCs and activated T cell blasts to help rule out a potential hypermorphic impact of the p.R77Q variant, although it would be unlikely due to the recessive mode of inheritance. The immunoblot analysis of PBMCs revealed that our patient had a slightly decreased IKKβ expression and normal IκBα levels compared to controls (**Figure 3A**). IKKβ and IκBα expression, however, was comparable to that of HDs in activated T cell blasts (**Figure 3A**). If there was a gain-of-function, we would expect a decrease in IκBα levels due to increased phosphorylation and degradation.

We additionally evaluated IKKβ activity by assessing IκBα degradation and p65 phosphorylation after NFkB pathway activation with TNFa. Both phospho-p65 and IκBα levels in patient activated T cells were comparable to that of the HDs following stimulation with TNFα. (**Figure 3B**, **Supplementary Figure 1**). Thus, the data does not support a possible hypermorphic nor hypomorphic function of the variant.

To further evaluate T cell function, we assessed the proliferative response of T cells to anti-CD2/CD3/CD28 beads after 3 or 5 days of culture. Our findings showed that patient and sibling-2 cells’ proliferative responses were comparable to HDs (**Figure 3C**, **Supplementary Figure 2**).

### 3.6 Functional characterization of the AID W80S substitution

**Figure 4:**
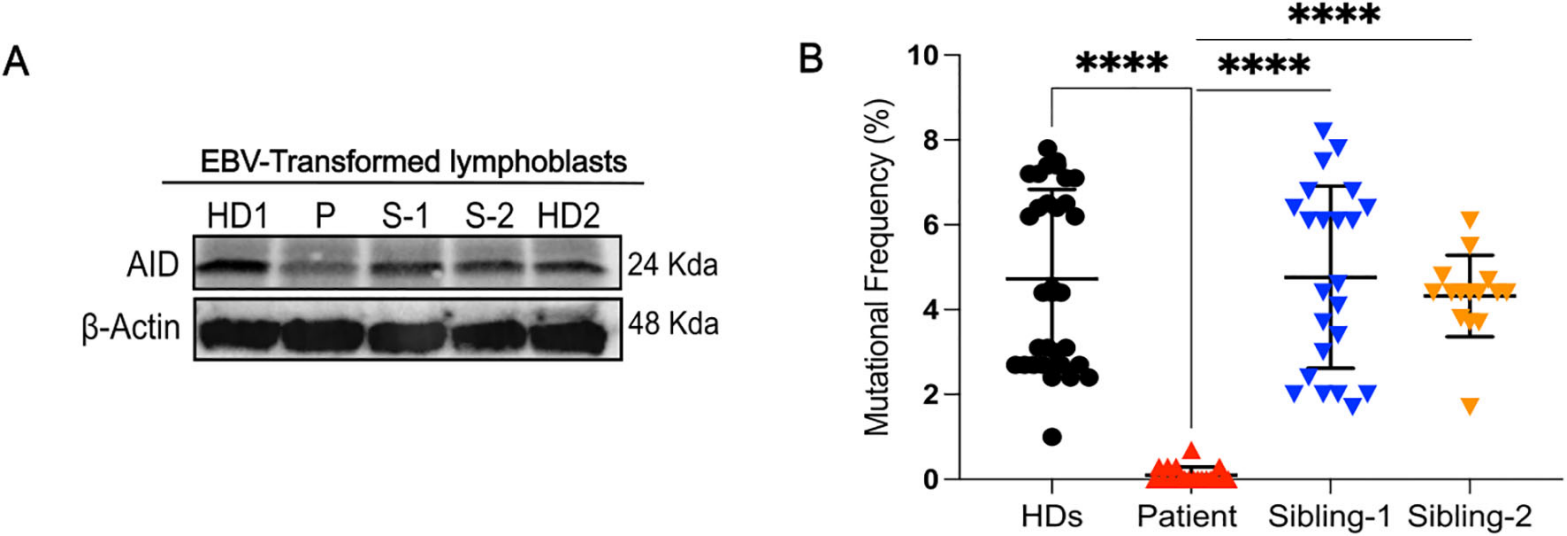
AID expression and analysis of somatic hypermutation. **(A)**, Western blot analysis for AID in EBV-transformed lymphoblasts from healthy donors (HDs), patient, and siblings (S-1 and S-2). **(B)**, Mutational frequency of the variable region of the IgM heavy chain of EBV lymphoblasts in different healthy donors (HDs) (32 clones from n=5 different controls), sibling-1 (23 clones), sibling-2 (15 clones) and patient (19 clones); each symbol represents one clone. Horizontal bars represent mean ± SD values of mutational frequency, Statistical analysis was performed using the Mann-Whitney U test; ****p<0.0001.

We examined the levels of the AID protein in the EBV-transformed human B-cell lines of sibling-1, sibling-2, patient, and HDs to confirm the *in silico* predicted destabilizing effect of the W80S AID mutation. The findings showed that the patient cells displayed much lower AID expression than did controls and the heterozygous siblings’ cells (**Figure 4A**). Transcript levels of *AICDA* were not reduced in the patient compared to controls (**Supplementary Figure 3**).

Therefore, we evaluated the functional impact of the W80S AID variant on SHM by comparing the mutational frequency within the variable region of the immunoglobulin heavy chain amplified from EBV-transformed B-cell lines from patient, siblings, and HDs. We found a near complete loss of SHM (0.10% ± 0.1915%) from the patient (**Figure 4B**, **Supplementary Table 1**).

### 3.7 ACMG classification

Variant classification was performed using the American College of Medical Genetics and Genomics (ACMG) and the Association for Molecular Pathology (AMP) guidelines 2015 (15), and criteria were applied based on genetic, functional, and clinical data. These ACMG/AMP standards provide a systematic framework for interpreting sequence variants using defined criteria, which help determine the clinical significance of genetic alterations. For the IKKβ p.R77Q variant, careful review of the clinical history of the homozygous sibling found that she is currently healthy, which is inconsistent with the known fully penetrant phenotype of IKKβ deficiency, and meets the BS2 criterion. The normal NF-κB functional results were in favor of the BS3 criterion, resulting in classification to likely Benign/Benign. In contrast, classification of the AID p.W80S variant included moderate evidence from domain localization (PM1), functional impairment (PS3), and a strong phenotype match (PP4), leading to a classification of likely pathogenic/pathogenic (**Table 2**).

**Table 2:** ACMG classification of *AICDA* and *IKBKB* variants.

Criterion	*AICDA* p.W80S	*IKBKB* p.R77Q
Population frequency (PM2)	Absent in gnomAD (PM2, moderate)	Absent in gnomAD (PM2, moderate)
*In silico* prediction (PP3)	Predicted damaging by CADD, PolyPhen-2, SIFT, MutationTaster, PrimateAI (PP3, supporting)	Conflicting predictions (PP3, partial supporting)
Located in critical domain (PM1)	In catalytic domain; known hotspot (PM1, moderate)	Outside kinase domain, no known hotspot (PM1 not applicable)
Functional evidence (PS3)	Reduced AID protein, defective SHM & CSR (PS3, strong)	Normal NF-κB activation (BS3)
Segregation/Inheritance (PM3/PP1)	Consistent with Autosomal Recessive (AR) pattern; both parents are carriers (PM3, moderate)	Inconsistent with expected fully penetrant IKKβ deficiency, currently healthy homozygous sibling is carrier (BS2)
Clinical phenotype match (PP4)	Clinical HIGM phenotype matches (PP4, supporting)	Phenotype partially fits but not classic IKKβ deficiency (PP4 not applicable)
Final Classification	Likely Pathogenic to Pathogenic	Likely Benign to Benign

ACMG, American College of Medical Genetics and Genomics; PM1, Pathogenic Moderate 1: The variant is found in a region of the gene known to be functionally important or frequently affected by disease-causing changes; PM2, Pathogenic Moderate 2: It is either completely absent or extremely rare in large population databases like gnomAD; PP1, Pathogenic supporting 1: The variant is inherited along with the disease in multiple affected members of the same family; PP3, Pathogenic supporting 3: Several bioinformatics tools predict that this change likely has a damaging effect; PP4, Pathogenic supporting 4: The patient’s symptoms or family history closely match specific monogenic disease; PS3, Pathogenic Strong 3: Laboratory experiments (functional studies) have shown that the variant has a negative impact on the gene function or its protein; BS2, Benign Strong 2: Found homozygous/hemizygous in healthy adults for recessive disorder or heterozygous for dominant disorder (15).

## 4 Discussion

Overlapping symptoms and biomarkers in well-known IEI is an emerging diagnostic and therapeutic challenge, which is further complicated by the identification of VUS, especially multiple VUS, during clinical genetic testing. In this article, we provide a comprehensive immunological and genetic study of a patient with two homozygous VUSs in the *IKBKB* and *AICDA* genes suspected to be responsible for the hyper-IgM and immune deficiency observed in a Tunisian patient treated at Sidra Medicine in Qatar.

To date only 7 nonsense (7, 16–21), 8 missense (22–29), and 2 duplication (11, 30) *IKBKB* mutations have been described. IKKβ deficient patients presented with life-threatening bacterial and viral infections, oral candidiasis, and failure to thrive (30). In addition to the episode of acute severe respiratory infection aged 2 years, our patient had an episode of oral thrush during infancy. Clinically, the patient also was found to have iron deficiency anemia and failure to thrive. Although opportunistic infections have been described in IKKβ deficient patients, it is unusual for AID deficiency (31). Thus, we considered IKKβ R77Q as a potential candidate along with the AID W80S variant, especially since sibling-2, who was also homozygous for the *IKBKB* variant, had a prior history of recurrent infections. Since IKKβ and AID deficiency are associated with different disease prognoses and therapeutic strategies, it was important to distinguish which disease, if not both, did the patient have. Multiple *in silico* tools indicated that the variants could potentially be deleterious and might impact the structure folding free energy. Both variants affected residues that were highly conserved between various species. *In silico* tools alone were insufficient to guide diagnosis, thus functional studies were performed.

The proband was found to exhibit a significant deficiency of class-switched CD27^+^IgM^-^IgD^-^ memory B cells and this is consistent with AID and IKKβ deficiencies, as a reduction in switched memory B cells is a feature of both diseases (7, 32). However, the normal level of total memory B cells is characteristic of AID deficiency but not IKKβ deficiency. Since T cell defects and deficiency of Tregs are a hallmark of previously reported homozygous *IKBKB* mutations in patients with severe early-onset immune deficiency (17, 30), we also examined T cell phenotype and function. Cardinez et al. (28) had previously reported that a hypermorphic variant of *IKBKB* had normal Treg and memory T cell subsets (28). In our patient, functional studies revealed no major impairments in T cell activation or proliferation, including comparable IκBα degradation and NF-κB p65 phosphorylation following stimulation. These results, together with normal IKKβ and IκBα levels in activated T cell blasts, argue against a gain- or loss-of-function mechanism. Taken together, the findings indicate that the IKKβ variant is unlikely to be disease-causative.

Of interest, AID deficiency is the most prevalent underlying molecular cause of immunoglobulin class-switch recombination defects in Tunisian individuals (68%) (33). The patient was diagnosed as having elevated IgM and decreased levels of the other immunoglobulin classes, which is indicative of a CSR defect (**Table 1**). According to earlier research, both CSR and SHM may be abolished by mutations in AID’s catalytic domain (14, 34), suggesting that W80 might be a key residue for AID enzymatic activity. Structurally, AID is a 198-amino-acid protein with a conserved catalytic core containing a zinc-binding motif necessary for the deamination of cytosine to uracil in single-stranded DNA (35). The N-terminal region aids in targeting immunoglobulin gene loci, while the C-terminal domain is crucial for interactions with DNA repair proteins such as UNG and MSH2/MSH6, which are particularly important for CSR (36, 37). The W80S mutation identified in our patient resides in exon 3, within the catalytic domain, a region with the highest degree of conservation (especially residue positions 75-87) and where the most damaging missense mutations per length have been reported (38). Tryptophan 80 is a conserved residue believed to contribute to structural stability and proper positioning of the catalytic site (39). Substitution with a polar residue like serine might disrupt the local hydrophobic environment, likely imparing AID activity. Our results indicate that the W80S mutation impairs both somatic hypermutation and class-switch recombination, resulting clinically in AID deficiency (40).

In a cohort of HIGM patients described by Revy et al. in 2000, a mutation causing the amino acid substitution of tryptophan (W) to arginine (R) at the same position (residue 80) was identified in a Turkish family with AID deficiency (2). Interestingly, the W80S mutation resulted in a reduced level of AID protein in the patient’s EBV-transformed lymphoblasts, in contrast to this previously reported p.W80R mutation, which exhibited a normal AID expression in transfected 293T cells (14). In fact, the *in silico* study of the ΔΔG values for both mutations supported the hypothesis that the W80S mutation may be more disruptive than the W80R variant. Importantly, the patient (P16) with the W80R variant had low serum IgG and IgA concentrations (14), whereas the patient described here with the W80S variant had IgG and IgA levels that were undetectable, thus further indicating that the W80S variant may be more damaging than W80R.

The previously reported pathogenic H56Y and C87R AID missense mutations, neighboring the W80 residue, affect conserved residues in the AID’s catalytic domain, yet they appear to differ in their biochemical and clinical consequences (41). A previous comprehensive structural analysis of AID mutations, was conducted by Mu et al. (41), in which the authors generated a structural model of AID using Apo3G-CD2 as a template and analyzed various mutations associated with Hyper-IgM syndrome type 2. They highlighted that residues such as H56 and C87 are involved in coordinating the zinc ion, which is crucial for AID’s catalytic activity. Mutations at these sites were shown to be important for catalysis and potentially disruptive to the enzyme’s function (41). The C87R mutation, located within the zinc-coordinating domain essential for AID’s catalytic function, has been associated with a severe immunodeficiency phenotype and complete loss of class-switch recombination (42). In contrast to our case, p.C87R transfected in 293T cells express normal amount of AID protein (14). H56Y was markedly reduced by Western blot, suggesting that most likely H56Y impairs protein stability or expression (14, 43).

Abolhassani et al. reported that 100% of missense mutations located in exon 3 of the *AICDA* gene led to fungal infections and susceptibility to cancer (38). These findings appear consistent with our patient’s experience with fungal infection and further suggest the need to closely monitor the patient for the development of cancer.

Given the consanguineous background and the patient’s Tunisian origin, the AID p.W80S variant could represent either an isolated mutation or a founder mutation. Although we did not perform haplotype analysis, the absence of this variant in large public databases such as gnomAD or in studies with Tunisian patients (33, 43, 44), suggests that it is extremely rare or potentially specific to some families. Consanguinity rates in Tunisia remain high, contributing to the expression of rare autosomal recessive mutations (up to 30–40%) (45, 46). Previous studies from Tunisian cohorts have highlighted the emergence of founder mutations in other immunodeficiencies, particularly in autosomal recessive forms of Hyper-IgM syndrome and other IEIs (33, 47–49). Future regional sequencing efforts could clarify whether the p.W80S mutation represents a founder or a novel unique variant in this population.

In summary, the patient’s clinical, laboratory, and immunological phenotype was consistent with a defect in both CSR and SHM and with the diagnosis of hyper-IgM syndrome type 2. The patient is now symptomatically better and continues to receive monthly IVIG replacement therapy. She is still being closely monitored.

This study highlights the challenges inherent in diagnosing immune deficiencies. The overlapping symptoms presented by many of these disorders complicate diagnosis, and the identification of multiple VUSs adds another layer of complexity. *In silico* predictions can provide valuable insights, however, they are insufficient on their own to guide diagnosis. Functional studies are needed to clarify the pathogenicity of VUSs identified in clinical genetic testing, which is important in order to provide a definitive diagnosis for treatment guidance, especially in consanguineous patients who may have multiple homozygous rare VUSs.

## Data Availability

The original contributions presented in the study are included in the article/**Supplementary Material**. Further inquiries can be directed to the corresponding author.
