# Supplementary material for: Dual variants of uncertain significance in a case of hyper-IgM syndrome: implications for diagnosis and management
Source: Front Immunol. 2025 Jun 2;16:1594636. doi: 10.3389/fimmu.2025.1594636 (PMC12171361; doi:10.3389/fimmu.2025.1594636)
Supplement: Supplementary file 1 [file DataSheet1.pdf]

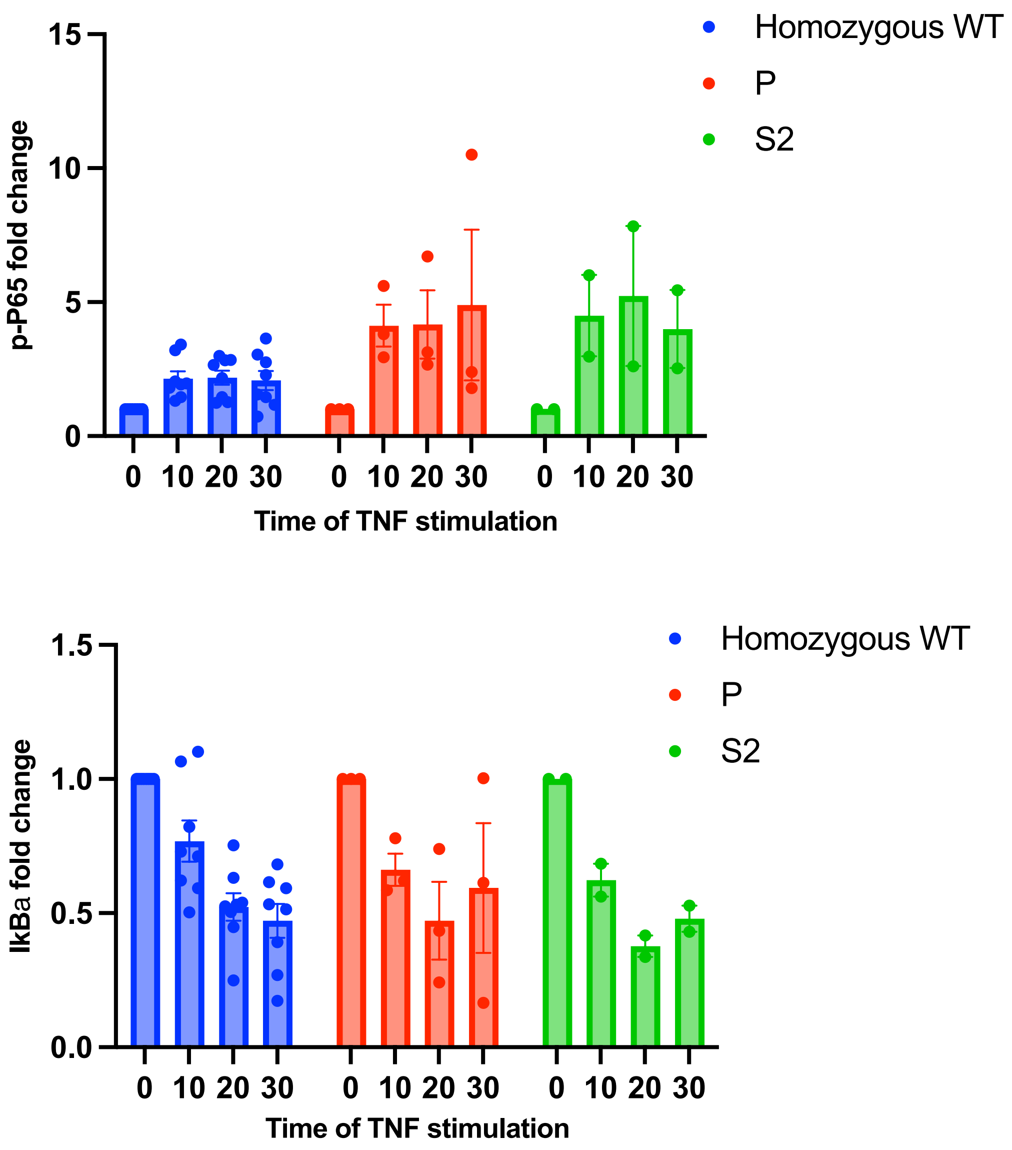

Supplementary Figure 1. Fold-change in the levels of phospho-p65 (top) and IkBα (bottom) at different time points (0-30min) post-TNFα stimulation. Data from patient (P) and healthy donors (including sibling 1), who are homozygous for WT *IKBKB*, are from 3 independent experiments, and data from sibling 2 (S2) are from 2 independent experiments. Two-way ANOVA multiple comparison was used for statistics. No significant difference between patient and homozygous WT controls were found.

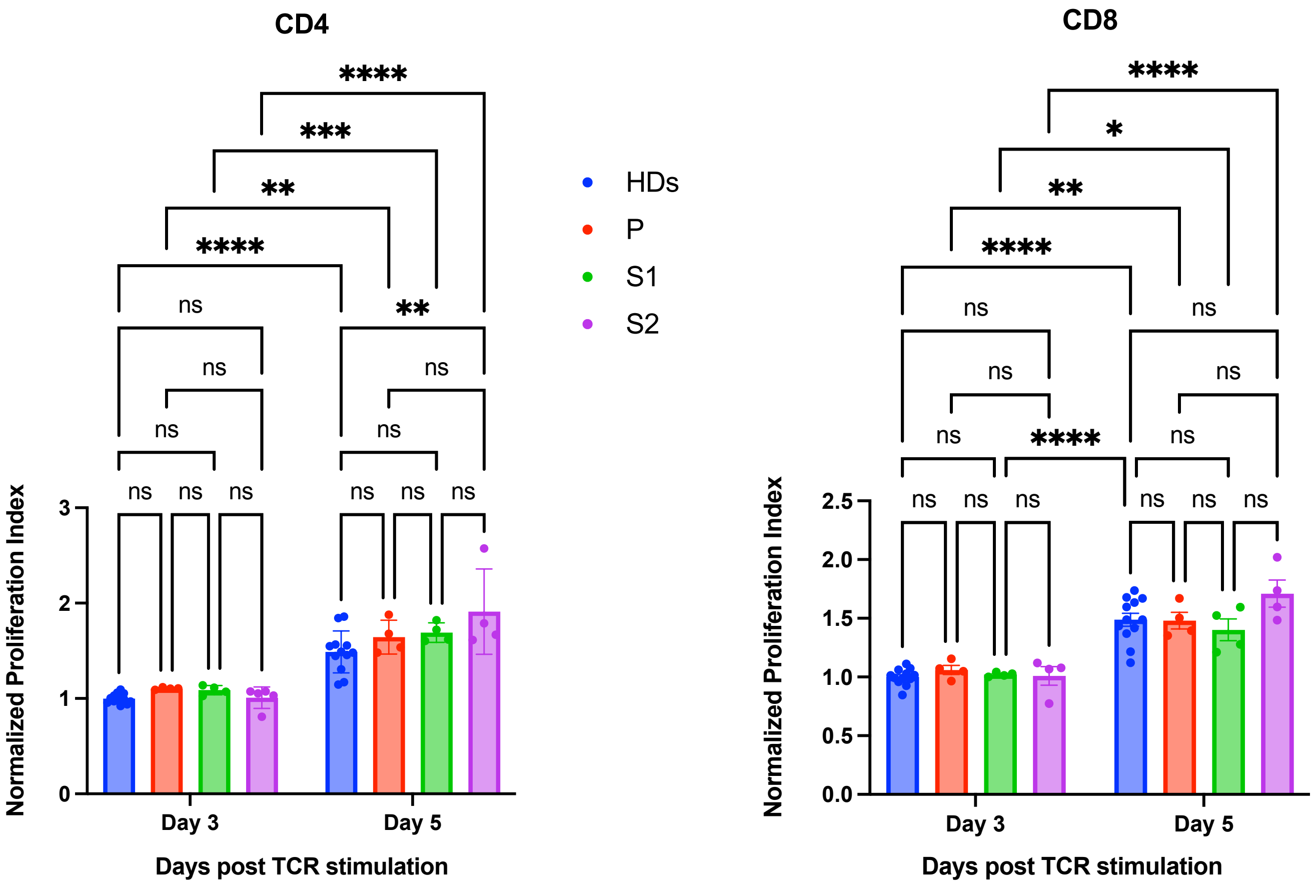

Supplementary Figure 2. PBMCs were stimulated for 3 days with beads coated with anti-CD3, anti-CD2 and anti-CD28. Cells were stained for viability and with antibodies against CD4 and CD8. Proliferation indexes were calculated usisng FlowJo proliferation tool and normalized to the average day 3 proliferation index of heathy donors (HDs). Two-way ANOVA multiple comparison was used for statistics. There was no significant difference in proliferation indices between patient (P), sibling 1 (S1), sibling 2 (S2), or healthy donors.

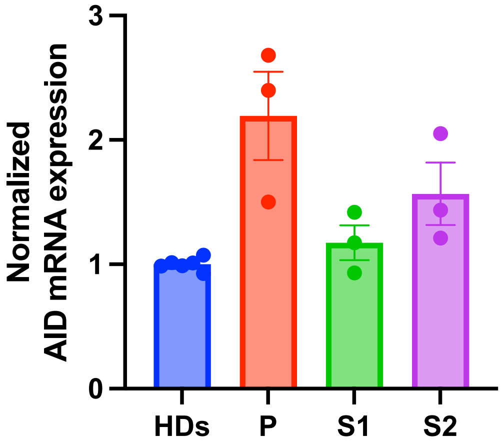

Supplementary Figure 3. qRT-PCR results of an experiment performed in technical triplicate. mRNA of AID is not reduced in the patient compared to healthy donors (HDs, n=2), sibling 1 (S1), or sibling 2 (S2) but rather appears to be increased. This suggests that the loss in protein expression is likely due to structural instability rather than an effect on transcription.
