# Supplementary material for: Dual variants of uncertain significance in a case of hyper-IgM syndrome: implications for diagnosis and management
Source: Front Immunol. 2025 Jun 2;16:1594636. doi: 10.3389/fimmu.2025.1594636 (PMC12171361; doi:10.3389/fimmu.2025.1594636)
Supplement: Supplementary file 2 [file Table1.docx]

**Supplementary Table 1: Mutation Frequency of Cloned IgH Sequences.**

| **Patient** | | | |
| --- | --- | --- | --- |
| Clone Number | Mismatches | % Mutational Frequency | % Identity |
| 1 | 0 | 0 | 100 |
| 2 | 0 | 0 | 100 |
| 3 | 0 | 0 | 100 |
| 4 | 1 | 0.3 | 99.7 |
| 5 | 1 | 0.3 | 99.7 |
| 6 | 0 | 0 | 100 |
| 7 | 0 | 0 | 100 |
| 8 | 0 | 0 | 100 |
| 9 | 0 | 0 | 100 |
| 10 | 0 | 0 | 100 |
| 11 | 0 | 0 | 100 |
| 12 | 0 | 0 | 100 |
| 13 | 2 | 0.7 | 99.3 |
| 14 | 0 | 0 | 100 |
| 15 | 1 | 0.3 | 99.7 |
| 16 | 0 | 0 | 100 |
| 17 | 1 | 0.3 | 99.7 |
| 18 | 0 | 0 | 100 |
| 19 | 0 | 0 | 100 |
| **Sibling-1** | | | |
| Clone Number | Mismatches | % Mutational Frequency | % Identity |
| 1 | 20 | 6.8 | 93.2 |
| 2 | 19 | 6.4 | 93.6 |
| 3 | 20 | 6.8 | 93.2 |
| 4 | 19 | 6.4 | 93.6 |
| 5 | 17 | 6.1 | 93.9 |
| 6 | 18 | 6.1 | 93.9 |
| 7 | 18 | 6.1 | 93.9 |
| 8 | 7 | 2.4 | 97.6 |
| 9 | 18 | 6.1 | 93.9 |
| 10 | 11 | 3.7 | 96.3 |
| 11 | 10 | 3.4 | 96.6 |
| 12 | 6 | 2 | 98 |
| 13 | 5 | 1.7 | 98.3 |
| 14 | 22 | 7.8 | 92.2 |
| 15 | 12 | 4.4 | 95.6 |
| 16 | 21 | 7.5 | 92.5 |
| 17 | 23 | 8.2 | 91.8 |
| 18 | 12 | 4.6 | 95.4 |
| 19 | 6 | 2 | 98 |
| 20 | 6 | 2 | 98 |
| 21 | 11 | 4.1 | 95.9 |
| 22 | 9 | 3 | 97 |
| 23 | 6 | 2 | 98 |
| **Sibling-2** | | | |
| Clone Number | Mismatches | % Mutational Frequency | % Identity |
| 1 | 13 | 4.4 | 95.6 |
| 2 | 12 | 4.4 | 95.6 |
| 3 | 13 | 4.4 | 95.6 |
| 4 | 11 | 3.7 | 96.3 |
| 5 | 13 | 4.4 | 95.6 |
| 6 | 11 | 3.7 | 96.3 |
| 7 | 13 | 4.4 | 95.6 |
| 8 | 17 | 6.1 | 93.9 |
| 9 | 15 | 5.5 | 94.5 |
| 10 | 13 | 4.4 | 95.6 |
| 11 | 14 | 4.8 | 95.2 |
| 12 | 14 | 4.7 | 95.3 |
| 13 | 5 | 1.7 | 98.3 |
| 14 | 13 | 4.4 | 95.6 |
| 15 | 11 | 3.8 | 96.2 |
| **Healthy Donors** | | | |
| Clone Number | Mismatches | % Mutational Frequency | % Identity |
| 1* | 21 | 7.1 | 92.9 |
| 2* | 22 | 7.4 | 92.6 |
| 3 | 7 | 2.4 | 97.6 |
| 4 | 19 | 6.5 | 93.5 |
| 5 | 19 | 6.4 | 93.6 |
| 6 | 13 | 4.5 | 95.5 |
| 7 | 22 | 7.5 | 92.5 |
| 8 | 9 | 3 | 97 |
| 9 | 8 | 2.7 | 97.3 |
| 10 | 19 | 6.4 | 93.6 |
| 11 | 11 | 4.4 | 95.6 |
| 12 | 12 | 4.4 | 95.6 |
| 13 | 21 | 7.2 | 92.8 |
| 14 | 18 | 7.2 | 92.8 |
| 15 | 7 | 2.4 | 97.6 |
| 16 | 9 | 3.1 | 96.9 |
| 17 | 8 | 2.7 | 97.3 |
| 18 | 18 | 6.2 | 93.8 |
| 19 | 7 | 2.4 | 97.6 |
| 20 | 8 | 2.7 | 97.3 |
| 21 | 8 | 2.7 | 97.3 |
| 22* | 3 | 1 | 99 |
| 23* | 13 | 4.4 | 95.6 |
| 24* | 21 | 7.1 | 92.9 |
| 25 | 9 | 3.1 | 96.9 |
| 26 | 9 | 3.1 | 96.9 |
| 27 | 7 | 2.7 | 97.3 |
| 28* | 21 | 7.4 | 92.6 |
| 29 | 8 | 2.7 | 97.3 |
| 30 | 19 | 6.5 | 93.5 |
| 31* | 23 | 7.8 | 92.2 |
| 32 | 18 | 6.2 | 93.8 |

*Clone from age-matched control
